# Otorhinolaryngologic complications after COVID-19 vaccination, vaccine adverse event reporting system (VAERS)

**DOI:** 10.3389/fpubh.2023.1338862

**Published:** 2024-01-10

**Authors:** Jieun Shin, Sung Ryul Shim, Jaekwang Lee, Hyon Shik Ryu, Jong-Yeup Kim

**Affiliations:** ^1^Department of Biomedical Informatics, College of Medicine, Konyang University, Daejeon, Republic of Korea; ^2^Konyang Medical data Research group-KYMERA, Konyang University Hospital, Daejeon, Republic of Korea; ^3^Department of Emergency Medicine, College of Medicine, Konyang University Hospital, Daejeon, Republic of Korea; ^4^Department of Otorhinolaryngology-Head and Neck Surgery, College of Medicine, Konyang University Hospital, Daejeon, Republic of Korea

**Keywords:** COVID-19 vaccines, drug-related side effects and adverse reactions, otolaryngological adverse events, COVID-19, vaccines

## Abstract

**Background:**

There have been reports of otolaryngological adverse event following immunization (AEFI) such as instances of olfactory and gustatory dysfunction following COVID-19 vaccination. This study aimed to analyze otolaryngological AEFIs following COVID-19 vaccination.

**Methods:**

This study was conducted with a secondary data analysis that the Vaccine Adverse Events Reporting System (VAERS) and the COVID-19 Data Tracker, which are both administered by the Centers for Disease Control and Prevention in the US. Using Medical Dictionary for Regulatory Activities (MedDRA) concepts, AEFIs included: Considering the overall frequency and similarity of symptoms in the first 153 PTs, they were grouped into major 19 AEFIs groups. The incidence rates (IRs) of AEFIs per 100,000 were calculated on individual and cumulative AEFIs levels, involving people who received complete primary series and an updated bivalent booster dose with one of the available COVID-19 vaccines in the US. The proportions of AEFIs by age, sex, and vaccine manufacturer were reported. We also calculated the proportional reporting ratio (PRR) of AEFIs.

**Results:**

We identified 106,653 otorhinolaryngologic AEFIs from the VAERS database, and a total of 226,593,618 people who received complete primary series in the US. Overall, the IR of total Otorhinolaryngologic AEFIs was 47.068 of CPS (completed primary series) and 7.237 UBB (updated bivalent booster) per 100,000. For most symptoms, being female was associated with statistically significant higher AEFIs. Upon examining the impact of different vaccine manufacturers, the researchers found that Janssen’s vaccine exhibited higher IRs for hearing loss (5.871), tinnitus (19.182), ear infection (0.709), dizziness (121.202), sinusitis (2.088), epistaxis (4.251), anosmia (5.264), snoring (0.734), allergies (5.555), and pharyngitis (5.428). The highest PRRs were for Anosmia (3.617), Laryngopharyngeal Reflux - Acid Reflux (2.632), and Tinnitus -Ringing in the ears (2.343), in that order, with these three significantly incidence than other background noises.

**Conclusion:**

This study, utilizing an extensive sample sizes, represents a significant step toward comprehensively characterizing the otolaryngological AEFIs associated with COVID-19 vaccinations. This large-scale analysis aims to move beyond isolated case reports and anecdotal evidence, providing a robust and detailed portrait of the otolaryngological AEFIs landscape in response to COVID-19 vaccinations.

## 1 Introduction

The COVID-19 pandemic, caused by the SARS-CoV-2 virus, has had a profound global impact, leading to significant morbidity and mortality rates (1). In response to this unprecedented health crisis, an intense global effort was made to develop vaccines to prevent COVID-19. In December 2020, the US Food and Drug Administration (FDA) granted Emergency Use Authorization (EUA) for the COVID-19 mRNA vaccine developed by Pfizer-BioNTech (2). Subsequent authorizations were granted for the mRNA-1273 vaccine developed by Moderna (3), and the Ad26.COV2 vector-based vaccine developed by Janssen Johnson & Johnson (3).

The EUAs issued by the FDA facilitated rapid deployment of these vaccines based on promising preliminary data, a pivotal decision considering the urgent need to curb the spread of the virus. However, this expedited the authorization process without extensive clinical trials typically required for full approval, thereby necessitating rigorous post-authorization safety monitoring.

Several case reports have been published detailing instances of olfactory and gustatory dysfunction following the COVID-19 vaccination (4, 5). However, the potential for broader otolaryngological adverse event following immunization (AEFI)—encompassing the ear, nose, and throat regions—associated with COVID-19 vaccination remains largely unexplored. According to the definition of the World Health Organization (WHO), AEFI is defined as any untoward medical occurrence following immunization which does not necessarily have a causal relationship to the vaccine. Given that these areas are frequent sites of viral infection and are also involved in immune responses, it is plausible that they may be vulnerable to AEFIs. Furthermore, the potential AEFIs associated with COVID-19 vaccination, studies have shown a waning immune response post-vaccination influenced by factors such as immunosenescence, gender-related hormonal differences, and pre-existing comorbidities (6–8).

To address this knowledge gap, this study conducted an analysis of otolaryngological AEFI reported after COVID-19 vaccination using the Vaccine VAERS data (9). The VAERS database is a national early-warning system designed to detect possible safety problems in US-licensed vaccines and plays a critical role in post-authorization safety monitoring (9).

This study aims to characterize the nature and prevalence of otolaryngological AEFIs with COVID-19 vaccines. The researchers further examined the demographic distribution of these AEFIs in terms of gender and age and evaluate the variation in these AEFIs among the different vaccine manufacturers (Pfizer-BioNTech, Moderna, and Janssen Johnson & Johnson). Ultimately, this study provides a basis for uncovering mechanisms and improving the understanding of the safety profile of COVID-19 vaccines through reporting of AEFIs following vaccination.

## 2 Materials and methods

This study followed the STROBE (Strengthening the Reporting of Observational Studies in Epidemiology) (10) reporting guidelines (Supplementary Table 1), and was conducted after receiving approval from the institutional review board of Konyang University (KYU-2023-09-002).

### 2.1 Study design

This study was conducted through secondary data analysis, collecting VAERS data from December 2020 to August 2023 to analyze otolaryngologic AEFIs associated with the COVID-19 vaccines authorized in the United States.

#### 2.1.1 Data source

The VAERS was developed in 1990 as a US vaccine safety surveillance program by the Centers for Disease Control and Prevention (CDC) and the Food and Drug Administration (FDA) (9). It collects information regarding adverse event (AE)s to serve as an early-warning system for potential safety issues regarding US-licensed vaccines. Vaccine recipients, health care providers, and vaccine makers can openly report side effects to VAERS (9). The VAERS data and individual reports without personally identifiable information were available to the public on the VAERS^1^ and CDC WONDER^2^ websites (all accessed through August 31, 2023). The details of the survey including the questionnaires, methodology, and description of the dataset were available on the aforementioned websites.

#### 2.1.2 Measurement

**Figure 1:**
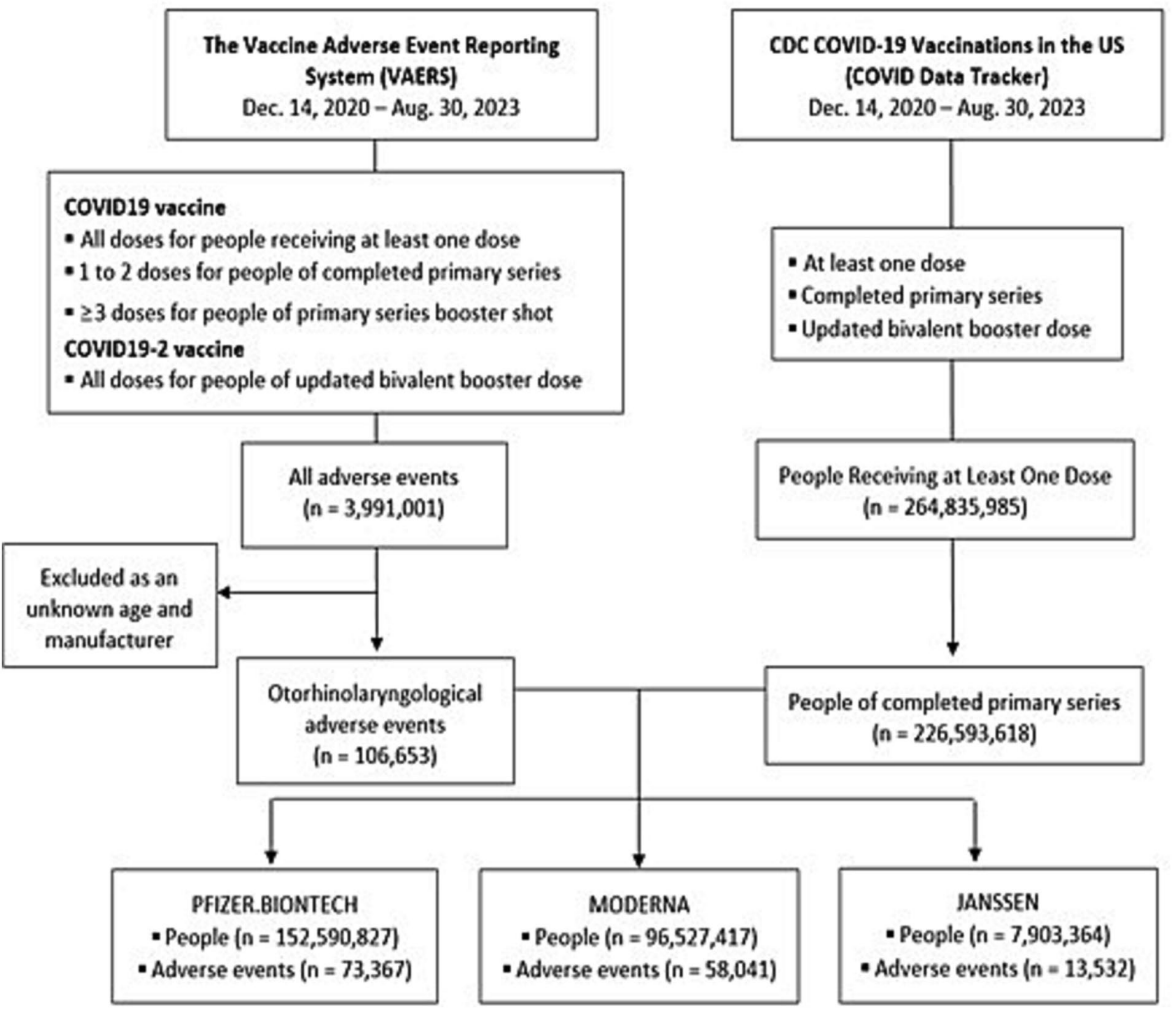
Flow diagram. People receiving completed primary series, the total number of people who received completed primary series of COVID-19 vaccine approved or authorized for use in the United States. Data are from the vaccine adverse event reporting system (VAERS) and CDC Covid-19 vaccinations data tracker from December 14, 2020 to August 30, 2023.

Since VAERS does not provide data on the entire vaccinated US population, the researchers used data from the CDC Data Tracker,^3^ which collected information from people who received complete primary series and an updated bivalent booster dose, by age, sex, and manufacturer. The CDC calculates rate and percentage in relation to vaccination among the entire population and selected demographic groups (e.g., individuals aged 65 or older). The data used for these calculations is from the US Census Bureau’s Annual Estimates of the Resident Population for the United States^4^ (Figure 1). The researchers then collected the reports of AEFIs incurred by 1 or 2 doses of the COVID19 vaccine, from people that received complete primary series. The AEFIs related to all number of doses of the COVID19-2 vaccine were collected from people who received an updated bivalent booster dose.

Age group was divided into five levels as: 0–17, 18–49, 50–64, and 64 or above using VAERS and CDC. The researchers compared the AEFIs incidence that incurred after the vaccination of the two mRNA vaccines (mRNA-1273, Moderna; and BNT162b2, Pfizer-BioNTech) or one viral vector vaccine (JNJ-78436735, Janssen/Johnson and Johnson), as reported in VAERS data. As the CDC did not provide the number of complete primary series of manufacturers, substituted the item with “At Least One Dose.” The CDC did not provide the number of updated bivalent booster made by Jassen because it was not used as an updated bivalent booster in the US.

#### 2.1.3 Adverse event

The otorhinolaryngologic AEFIs following the COVID-19 vaccination were based on the Medical Dictionary for Regulatory Activities (MedDRA) concepts at the preferred term (PT) level (11). In this study, 153 PTs were considered to be related to otolaryngology AEFIs through a meeting of otolaryngologists and all researchers (Supplementary Table 2). Considering the overall frequency and similarity of symptoms in the first 153 PTs, they were grouped into major 19 AEFIs groups (Supplementary Table 3).

Two researchers (JY Kim and JE Shin) independently screened the descriptions in the database to ensure the reliability of the Otorhinolaryngologic PTs. One author (JY Kim), a specialist in otorhinolaryngology, confirmed the retrieved terms and term groupings. The authors also examined all narrative text of coexisting current illnesses and comorbidities in VAERS. If they disagreed with the judgment of the description, the final PTs were determined by consensus of the researchers.

#### 2.1.4 Analyses of PRR

The proportional reporting ratio (PRR) is a commonly used method to assess the significance of AEFIs. It is a fundamental measure of disproportionality utilized by the FDA for data mining in the FAERS database (12), which analyzes drug-related data, including COVID-19 vaccines (13). To calculate the PRR, the ratio of the total cases for a specific AEFI associated with COVID-19 vaccines is divided by the ratio of the same AEFI for all other vaccines in the VAERS database. This calculation is akin to determining the relative risk of a drug. The PRR formula is as follows:


$$PPR=\frac{m}{n}\left[\frac{M-m}{N-n}\right]$$

m represents the number of cases for the specific AEFI of the COVID-19 vaccines.

n represents the total number of AEFI of the COVID-19 vaccines.

M represents the total number of cases for the specific AEFI in the VAERS database.

N represents the total number of all AEFI in the VAERS database.

The PRR serves as a valuable tool in evaluating the potential significance of AEFIs associated with COVID-19 vaccines and other drugs. A value of ≥2 indicates a signal that is greater than background noise (14–16).

### 2.2 Statistical analysis

The incidence rates (IRs) of AEFIs per 100,000 were calculated on individual and cumulative AEFIs levels, involving people who received complete primary series and an updated bivalent booster dose with one of the available COVID-19 vaccines in the US. The proportions of AEFIs by age, sex, and vaccine manufacturer were reported. Pearson’s chi-squared tests or Fisher’s exact tests were carried out to determine statistically significant differences between categories. The importance of AEFIs was assessed by calculating PRR.

All statistics were two-tailed, and *p* values <0.05 were considered statistically significant. R version 4.3.1 was used for all statistical analyses (R Foundation for Statistical Computing, Vienna, Austria).

## 3 Results

### 3.1 Characteristics of the study sample

The initial search identified a total of 106,653 otorhinolaryngologic AEFIs from the VAERS database, and a total of 226,593,618 people who received complete primary series in the US, based on the CDC Data Tracker between January 1, 2020 and August 30, 2023. Since the COVID-19 vaccine was first approved in the United States in December 2020, actual data were collected from December 2020 to August 2023. Of those reporting AEFIs, the number of AE reports from Pfizer-BioNTech, Moderna, and Janssen groups were 73,367 (50.6%), 58,041 (40.0%), and 13,532 (9.3%), respectively (Figure 1).

### 3.2 Comparison of AEFIs by sex group

**Figure 2:**
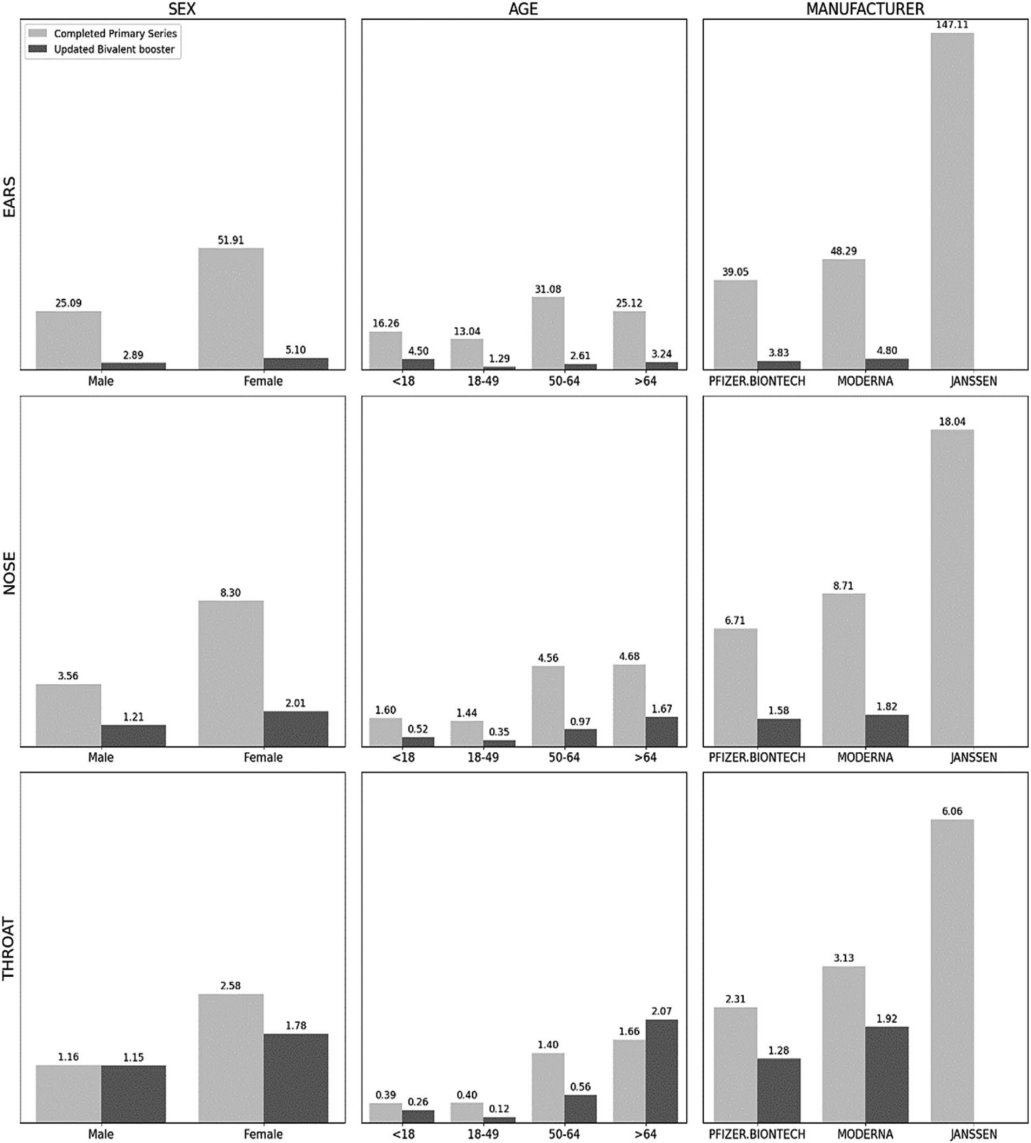
Incidence rates of adverse events by sex, age, and manufacturers. Note: Per 100,000 persons. Data are from the vaccine adverse event reporting system (VAERS) and CDC Covid-19 vaccinations data tracker from December 14, 2020 to August 30, 2023. In manufacturer, the CDC did not provide the number of Completed Primary Series, so we substituted At Least One dose. Ears (Hearing Loss, Tinnitus, Ear Infections, Meniere’s Disease, Vestibular Neuronitis, Dizziness or Vertigo); Nose (Sinusitis, Rhinitis, Epistaxis, Anosmia, Nasal Polyps, Snoring or Difficulty Breathing through the Nose and Sleep Apnea, Allergies); Throat (Tonsillitis, Laryngitis, Vocal Cord Polyps and Nodules, Laryngopharyngeal Reflux, Epiglottitis, Pharyngitis). All adverse events by sex, age, and manufacturer have statistically significant differences between categories using χ2-test or Fisher’s exact test.

The IRs of AEFI types per 100,000 people who received complete primary series with COVID-19 vaccines are presented in Figure 2 and Table 1. Overall, the IR of total Otorhinolaryngologic AEFIs was 47.068 of CPS (completed primary series) and 7.237 UBB (updated bivalent booster) per 100,000. For most symptoms, being female was associated with statistically significant higher AEFIs (Table 1).

**Table 1:** Otorhinolaryngologic adverse effects of COVID-19 vaccination in the United States.

Adverse effects	Completed primary series						Updated bivalent booster							
	Sum		Male		Female		*P* value	Sum		Male		Female		*p* value
	n(%)	IR	n(%)	IR	n(%)	IR		n(%)	IR	n(%)	IR	n(%)	IR	
Hearing loss	4,319(4.05)	1.906	1,759(5.46)	1.629	2,560(3.44)	2.158	<.0.001	19(64.86)	0.352	70(5.3)	0.278	126(4.65)	0.413	0.413
Tinnitus (Ringing in the ears)	12,338(11.57)	5.445	4,960(15.41)	4.593	7,378(9.91)	6.221	<.0.001	381(9.45)	0.684	146(11.05)	0.579	235(8.67)	0.771	0.771
Ear infections (Otitis Media)	563(0.53)	0.248	187(0.58)	0.173	376(0.5)	0.317	<.0.001	81(2.01)	0.145	24(1.82)	0.095	57(2.1)	0.187	0.187
Meniere’s disease	82 (0.08)	0.036	25(0.08)	0.023	57(0.08)	0.048	0.002	3(0.07)	0.005	1(0.08)	0.004	2(0.07)	0.007	0.007
Vestibular neuronitis	98 (0.09)	0.043	35(0.11)	0.032	63(0.08)	0.053	0.018	11(0.27)	0.02	4(0.3)	0.016	7(0.26)	0.023	0.023
Dizziness or vertigo	71,255(66.81)	31.446	20,126(62.53)	18.637	51,129(68.66)	43.108	<0.001	1,610(39.94)	2.89	483(36.56)	1.915	1,127(41.59)	3.697	<0.001
Sinusitis	1,333(1.25)	0.588	346(1.07)	0.32	987(1.33)	0.832	<0.001	270(6.7)	0.485	80(6.06)	0.317	190(7.01)	0.623	<0.001
Rhinitis (Allergic and Non-allergic)	126(0.12)	0.056	49(0.15)	0.045	77(0.1)	0.065	0.049	8(0.2)	0.014	2(0.15)	0.008	6(0.22)	0.02	0.02
Epistaxis	2,085(1.95)	0.92	700(2.17)	0.648	1,385(1.86)	1.168	<0.001	45(1.12)	0.081	15(1.14)	0.059	30(1.11)	0.098	0.098
Anosmia	3,652(3.42)	1.612	1,237(3.84)	1.146	2,415(3.24)	2.036	<0.001	227(5.63)	0.408	64(4.84)	0.254	163(6.01)	0.535	<0.001
Nasal polyps	15(0.01)	0.007	7(0.02)	0.006	8(0.01)	0.007	0.939	2(0.05)	0.004	1(0.08)	0.004	1(0.04)	0.003	0.003
Snoring or difficulty breathing through the nose and sleep apnea	494(0.46)	0.218	284(0.88)	0.263	210(0.28)	0.177	<0.001	152(3.77)	0.273	84(6.36)	0.333	68(2.51)	0.223	0.223
Allergies	5,983(5.61)	2.64	1,219(3.79)	1.129	4,764(6.4)	4.017	<0.001	213(5.28)	0.382	58(4.39)	0.23	155(5.72)	0.508	<0.001
Tonsillitis	73(0.07)	0.032	20(0.06)	0.019	53(0.07)	0.045	0.001	4(0.1)	0.007	1(0.08)	0.004	3(0.11)	0.01	0.01
Laryngitis	135(0.13)	0.06	23(0.07)	0.021	112(0.15)	0.094	<0.001	54(1.34)	0.097	11(0.83)	0.044	43(1.59)	0.141	0.141
Vocal cord polyps and nodules	27(0.03)	0.012	6(0.02)	0.006	21(0.03)	0.018	0.008	0	0	0	0	0	0	
Laryngopharyngeal reflux (acid reflux)	337(0.32)	0.149	43(0.13)	0.04	294(0.39)	0.248	<0.001	21(0.52)	0.038	6(0.45)	0.024	15(0.55)	0.049	0.049
Epiglottitis	8(0.01)	0.004	3(0.01)	0.003	5(0.01)	0.004	0.565	0	0	0	0	0	0	
Pharyngitis	3,730(3.5)	1.646	1,159(3.6)	1.073	2,571(3.45)	2.168	<0.001	753(18.68)	1.352	271(20.51)	1.075	482(17.79)	1.581	<0.001
Any IR	106,653	47.068	32,188	29.807	74,465	62.783	<0.001	4,031	7.237	1,321	5.238	2,710	8.89	<0.001
Sample size	226,593,618		107,987,092		118,606,526			55,703,085		25,218,543		30,484,542		

The data was collected from the VAERS and the CDC Covid-19 vaccinations data tracker as of August 30, 2023. The Incidence Rate(IR) per 100,000 was measured based on subjects with complete primary series vaccination and updated bivalent booster of COVID-19 vaccinations in the US. The sample size was from the CDC Data Tracker. *P* value was by chi-square, which tests the difference in AEs distribution according to age group.

In CPS, females showed a higher IR of hearing loss (2.158), tinnitus (6.221), ear infections (0.317), and dizziness with a notable IR of 43.108 for dizziness in the ear region. In the nasal region, epistaxis (1.168), anosmia (2.036), snoring (0.177), and allergies (4.017) were higher IR among females, while in the throat area, females were more likely to experience laryngitis (0.094), laryngopharyngeal reflux (0.248), and pharyngitis (2.168).

The UBB dataset similarly demonstrated higher IRs for dizziness (3.697), sinusitis (0.623), anosmia (0.535), allergies (0.508), and pharyngitis (1.581) among females.

### 3.3 Comparison of AEFIs by age group

The CPS dataset revealed varying age-based trends for different otolaryngological AEFIs in Figure 2 and Table 2. For symptoms related to the ear, the 50–64 age group demonstrated the highest IR of hearing loss (1.678), tinnitus (6.417), ear infections (0.186), and dizziness (22.715). Anosmia (1.174) also recorded the highest IR in the 50–64 age group. Conversely, the 65 and older age group showed the highest IR for sinusitis (0.557), rhinitis (0.051), epistaxis (0.682), snoring (0.257), and allergies (1.956). In the throat region, laryngitis (0.068) and laryngopharyngeal reflux (0.157) were most common among the 50–64 age group, whereas pharyngitis (1.505) was most prevalent among those 65 and older.

**Table 2:** Otorhinolaryngologic adverse effects of COVID-19 vaccination by age.

Adverse effects	Completed primary series									Updated bivalent booster								
	0–17 years		18–49 years		50–64 years		65+ years		*P* value	0–17 years		18–49 years		50–64 years		65+ years		*P* value
	n(%)	IR	n(%)	IR	n(%)	IR	n(%)	IR		n(%)	IR	n(%)	IR	n(%)	IR	n(%)	IR	
Hearing loss	155(3.79)	0.692	429(2.94)	0.437	885(4.53)	1.678	804(5.01)	1.576	<0.001	3(1.85)	0.098	17(6.32)	0.111	36(6.37)	0.264	79(4.83)	0.338	<0.001
Tinnitus (Ringing in the ears)	166(4.06)	0.741	724(4.96)	0.738	3,384(17.33)	6.417	2,056(12.8)	4.029	<0.001	9(5.56)	0.294	12(4.46)	0.078	81(14.34)	0.593	109(6.67)	0.466	<0.001
Ear infections (Otitis Media)	27(0.66)	0.121	49(0.34)	0.05	98(0.5)	0.186	76(0.47)	0.149	<0.001	5(3.09)	0.163	7(2.6)	0.046	21(3.72)	0.154	26(1.59)	0.111	<0.001
Meniere’s disease	1(0.02)	0.004	2(0.01)	0.002	19(0.1)	0.036	20(0.12)	0.039	1	0	0	1(0.37)	0.007	0	0	2(0.12)	0.009	1
Vestibular neuronitis	2(0.05)	0.009	10(0.07)	0.01	24(0.12)	0.046	15(0.09)	0.029	<0.001	1(0.62)	0.033	1(0.37)	0.007	2(0.35)	0.015	6(0.37)	0.026	0.672
Dizziness or vertigo	3,291(80.5)	14.695	11,582(79.32)	11.799	11,978(61.33)	22.715	9,849(61.33)	19.3	<0.001	120(74.07)	3.915	159(59.11)	1.039	217(38.41)	1.589	537(32.86)	2.294	<0.001
Sinusitis	21(0.51)	0.094	76(0.52)	0.077	265(1.36)	0.503	284(1.77)	0.557	<0.001	3(1.85)	0.098	5(1.86)	0.033	32(5.66)	0.234	132(8.08)	0.564	<0.001
Rhinitis (Allergic and Non-allergic)	2(0.05)	0.009	13(0.09)	0.013	24(0.12)	0.046	26(0.16)	0.051	<0.001	0	0	2(0.74)	0.013	2(0.35)	0.015	3(0.18)	0.013	0.978
Epistaxis	127(3.11)	0.567	247(1.69)	0.252	333(1.71)	0.631	348(2.17)	0.682	<0.001	5(3.09)	0.163	4(1.49)	0.026	4(0.71)	0.029	18(1.1)	0.077	<0.001
Anosmia	53(1.3)	0.237	372(2.55)	0.379	753(3.86)	1.428	599(3.73)	1.174	<0.001	0	0	4(1.49)	0.026	39(6.9)	0.286	98(6)	0.419	<0.001
Nasal Polyps	0	0	2(0.01)	0.002	3(0.02)	0.006	4(0.02)	0.008	0.387	0	0	0	0	1(0.18)	0.007	1(0.06)	0.004	0.875
Snoring or difficulty breathing through the nose and sleep apnea	8(0.2)	0.036	36(0.25)	0.037	64(0.33)	0.121	131(0.82)	0.257	<0.001	1(0.62)	0.033	5(1.86)	0.033	21(3.72)	0.154	71(4.35)	0.303	<0.001
Allergies	147(3.6)	0.656	664(4.55)	0.676	963(4.93)	1.826	998(6.21)	1.956	<0.001	7(4.32)	0.228	34(12.64)	0.222	33(5.84)	0.242	67(4.1)	0.286	<0.001
Tonsillitis	3(0.07)	0.013	22(0.15)	0.022	11(0.06)	0.021	6(0.04)	0.012	0.607	0	0	2(0.74)	0.013	0	0	0	0	0.262
Laryngitis	1(0.02)	0.004	10(0.07)	0.01	36(0.18)	0.068	28(0.17)	0.055	<0.001	0	0	3(1.12)	0.02	8(1.42)	0.059	32(1.96)	0.137	<0.001
Vocal cord polyps and nodules	0	0	1(0.01)	0.001	10(0.05)	0.019	6(0.04)	0.012	0.001	0	0	0	0	0	0	0	0	
Laryngopharyngeal reflux (Acid Reflux)	3 (0.07)	0.013	26(0.18)	0.026	83(0.43)	0.157	39(0.24)	0.076	<0.001	0	0	1(0.37)	0.007	1(0.18)	0.007	9(0.55)	0.038	0.127
Epiglottitis	0	0	1(0.01)	0.001	2(0.01)	0.004	1(0.01)	0.002	0.745	0	0	0	0	0	0	0	0	
Pharyngitis	81 (1.98)	0.362	335(2.29)	0.341	594(3.04)	1.126	768(4.78)	1.505	<0.001	8(4.94)	0.261	12(4.46)	0.078	67 (11.86)	0.491	444(27.17)	1.897	<0.001
Any IR	4,088	18.253	14,601	14.875	19,529	37.035	16,058	31.467	<0.001	162	5.285	269	1.758	565	4.138	1,634	6.981	<0.001
Sample size	22,396,020		98,160,420		52,731,727		51,031,000			3,065,181		15,303,884		13,654,874		23,407,228		

The data was collected from the VAERS and the CDC Covid-19 vaccinations data tracker as of August 30, 2023. The Incidence Rate(IR) per 100,000 was measured based on subjects with complete primary series vaccination and updated bivalent booster of COVID-19 vaccinations in the US. The sample size was from the CDC Data Tracker. *P* value was by chi-square, which tests the difference in AEs distribution according to age group.

In the UBB dataset, the highest IRs for tinnitus (0.593) and ear infections (0.154) were observed in the 50–64 age group, while dizziness (2.294), sinusitis (0.564), anosmia (0.419), snoring (0.303), allergies (0.286), and pharyngitis (1.897) were more frequent among those aged 65 and older.

### 3.4 Comparison of AEFIs by vaccine manufacturer

Upon examining the impact of different vaccine manufacturers, the researchers found that Janssen’s vaccine exhibited higher IRs for hearing loss (5.871), tinnitus (19.182), ear infection (0.709), dizziness (121.202), sinusitis (2.088), epistaxis (4.251), anosmia (5.264), snoring (0.734), allergies (5.555), and pharyngitis (5.428) when compared to other vaccines in the “At Least One Dose” analysis in Figure 2 and Table 3.

**Table 3:** Otorhinolaryngologic adverse effects of COVID-19 vaccination by manufacturer.

Adverse effects	At least one dose							Updated Bivalent booster				
	PFIZER.BIONTECH		MODERNA		JANSSEN		*P* value	PFIZER.BIONTECH		MODERNA		*P* value
	n(%)	IR	n(%)	IR	n(%)	IR		n(%)	IR	n(%)	IR	
Hearing loss	3,391(4.62)	2.222	2,455(4.23)	2.543	464(3.43)	5.871	<0.001	113(4.76)	0.319	85(4.96)	0.423	0.047
Tinnitus (Ringing in the ears)	9,308(12.69)	6.100	7,049(12.14)	7.303	1,516(11.2)	19.182	<0.001	226(9.52)	0.637	168(9.8)	0.837	0.007
Ear infections (Otitis Media)	453(0.62)	0.297	345(0.59)	0.357	56(0.41)	0.709	<0.001	51(2.15)	0.144	37(2.16)	0.184	0.248
Meniere’s disease	61(0.08)	0.040	50(0.09)	0.052	7(0.05)	0.089	1.000	2(0.08)	0.006	1(0.06)	0.005	0.920
Vestibular neuronitis	89(0.12)	0.058	66(0.11)	0.068	5(0.04)	0.063	0.619	6(0.25)	0.017	5(0.29)	0.025	0.520
Dizziness or vertigo	46,290(63.09)	30.336	36,646(63.14)	37.964	9,579(70.79)	121.202	<0.001	961(40.5)	2.709	668(38.95)	3.328	<0.001
Sinusitis	1,029(1.4)	0.674	941(1.62)	0.975	165(1.22)	2.088	<0.001	154(6.49)	0.434	117(6.82)	0.583	0.016
Rhinitis (Allergic and Non-allergic)	91(0.12)	0.060	83(0.14)	0.086	9(0.07)	0.114	0.020	4(0.17)	0.011	4(0.23)	0.020	0.414
Epistaxis	1,495(2.04)	0.980	1,072(1.85)	1.111	336(2.48)	4.251	<0.001	25(1.05)	0.070	20(1.17)	0.100	0.246
Anosmia	2,919(3.98)	1.913	2,032(3.5)	2.105	416(3.07)	5.264	<0.001	128(5.39)	0.361	101(5.89)	0.503	0.012
Nasal polyps	8(0.01)	0.005	6(0.01)	0.006	3(0.02)	0.038	0.002	1(0.04)	0.003	1(0.06)	0.005	0.683
Snoring or difficulty breathing through the nose and sleep apnea	568(0.77)	0.372	357(0.62)	0.370	58(0.43)	0.734	<0.001	121(5.1)	0.341	31(1.81)	0.154	<0.001
Allergies	4,134(5.63)	2.709	3,918(6.75)	4.059	439(3.24)	5.555	<0.001	126(5.31)	0.355	91(5.31)	0.453	0.075
Tonsillitis	50(0.07)	0.033	36(0.06)	0.037	8(0.06)	0.101	0.008	3(0.13)	0.008	1(0.06)	0.005	0.643
Laryngitis	135(0.18)	0.088	109(0.19)	0.113	15(0.11)	0.190	0.007	31(1.31)	0.087	24(1.4)	0.120	0.247
Vocal cord polyps and nodules	29(0.04)	0.019	13(0.02)	0.013	3(0.02)	0.038	0.225	(0)	0.000	(0)	0.000	
Laryngopharyngeal Reflux (Acid Reflux)	235(0.32)	0.154	193(0.33)	0.200	22(0.16)	0.278	0.002	13(0.55)	0.037	8(0.47)	0.040	0.852
Epiglottitis	6(0.01)	0.004	7(0.01)	0.007	2(0.01)	0.025	0.041	(0)	0.000	(0)	0.000	
Pharyngitis	3,076(4.19)	2.016	2,663(4.59)	2.759	429(3.17)	5.428	<0.001	408(17.19)	1.150	353(20.58)	1.759	<0.001
Any IR	73,367	48.081	58,041	60.129	13,532	171.218	<0.001	2,373	6.689	1,715	8.544	<0.001
Sample size	152,590,827		96,527,417		7,903,364			35,476,628		20,072,000		

The data was collected from the VAERS and the CDC Covid-19 vaccinations data tracker as of August 30, 2023. The Incidence Rate(IR) per 100,000 was measured based on subjects with complete primary series vaccination and updated bivalent booster of COVID-19 vaccinations in the US. The sample size was from the CDC Data Tracker. *P* value by Chi-square test. The CDC did not provide the number of the complete primary series, thus it was substituted with “At Least One Dose.” The CDC did not provide the number of updated bivalent booster produced by Jassen because it was not used as one in the US.

In the UBB group, higher IRs for dizziness (3.328) and pharyngitis (1.759) were observed for the Moderna vaccine compared to the Pfizer-BioNTech vaccine. Conversely, Pfizer-BioNTech exhibited a higher IR for snoring (0.341) compared to Moderna.

### 3.5 Proportional reporting ratio compared with other AEFIs

The highest PRRs were for Anosmia (3.617), Laryngopharyngeal Reflux - Acid Reflux (2.632), and Tinnitus -Ringing in the ears (2.343), in that order, with these three significantly incidence than other background noises (PRR >2) in Table 4. Hearing Loss(PRR:1.554), Ear Infectios (Otitis Me-dia; PRR:0.227), Meniere’s Disease (PRR:1.945), Dizziness or Vertigo (PRR:1.629), Sinusitis (PRR:0. 832), Rhinitis (Allergic and Non-allergic; PRR:0.056), Epistaxis (PRR:1.605), Snoring or Difficulty Breathing through the Nose and Sleep Ap-nea (PRR:0. 205), Allergies (PRR:0.251), Tonsillitis (PRR:0.491), Layryngitis (PRR:0.332), Epiglottitis (PRR:0.348), Pharyngitis (0.573) were statistically significant but did not show clinically significant incidence when compared to other AEFIs (PRR <2; Table 4).

**Table 4:** Proportional reporting ratios in completed primary series.

Symptoms	Completed primary series			
	Specific AEs of COVID-19 vaccines	PRR	95% CIL	95% CIH
Hearing loss	4,319	1.554	1.487	1.625
Tinnitus (Ringing in the ears)	12,338	2.343	2.275	2.413
Ear infections (Otitis Media)	563	0.227	0.207	0.248
Meniere’s disease	82	1.945	1.382	2.736
Vestibular neuronitis	98	1.217	0.924	1.603
Dizziness or vertigo	71,255	1.629	1.612	1.647
Sinusitis	1,333	0.832	0.777	0.891
Rhinitis (Allergic and Non-allergic)	126	0.056	0.047	0.067
Epistaxis	2,085	1.605	1.506	1.712
Anosmia	3,652	3.167	2.983	3.363
Nasal polyps	15	1.956	0.879	4.355
Snoring or difficulty breathing through the nose and sleep apnea	494	0.205	0.186	0.225
Allergies	5,983	0.251	0.244	0.258
Tonsillitis	73	0.491	0.375	0.642
Laryngitis	135	0.332	0.275	0.401
Vocal cord polyps and nodules	27	1.101	0.659	1.837
Laryngopharyngeal reflux (Acid Reflux)	337	2.632	2.187	3.168
Epiglottitis	8	0.348	0.159	0.759
Pharyngitis	3,730	0.573	0.551	0.596
Any IR	106,653			
Sample size	226,593,618			

Data are from the VAERS and CDC Covid-19 vaccinations data tracker through to August 30, 2023. PRR, proportional reporting ratio.

## 4 Discussion

In the context of the global rollout of COVID-19 vaccinations, understanding potential AEFIs is of paramount importance. Previous studies have mainly focused on general systemic or localized AEs, leaving otolaryngological AEFIs relatively unexplored (17–19). This study, utilizing an extensive sample size of 226,593,618 individuals, represents a significant step toward comprehensively characterizing the otolaryngological AEFIs associated with COVID-19 vaccinations. This large-scale analysis aims to move beyond isolated case reports and anecdotal evidence, providing a robust and detailed portrait of the otolaryngological AEFIs landscape in response to COVID-19 vaccinations.

One of the most salient findings from the study was the high incidence of dizziness/vertigo as an otolaryngological AEFIs post COVID-19 vaccination. This observation aligns with prior literature, notably the research conducted by Yan et al., which too highlighted a significant increase in episodes of dizziness/vertigo subsequent to COVID-19 vaccination (20). Drawing from the detailed assessment by Yan et al., it is interesting to note that the time to the onset of these symptoms post-vaccination was approximately 10 days, coinciding with the onset of IgG production. This suggests a potential immunological underpinning for the manifestation of these symptoms. Furthermore, their research emphasized the exacerbation of conditions such as Meniere’s disease (MD) post-vaccination, potentially due to heightened immunological factors leading to aggravated endolymphatic hydrops (21). Other conditions such as Vertebrobasilar insufficiency (VBI) were also implicated, pointing to dysregulation of blood flow and factors such as altered plasma viscosity post-vaccination. Notably, while some vaccines, like the AstraZeneca (AZ) variant, demonstrated efficacy against SARS-COV-2, they were associated with a heightened risk of thrombotic events (22). Finally, it is essential to consider the backdrop against which these vaccinations are taking place. The ongoing stress and heightened anxiety levels during this pandemic might contribute to immunization anxiety-related reactions. Therefore, while this study and others highlight significant otolaryngological AEFIs, it underscores the need for a comprehensive understanding and approach toward managing post-vaccination AEFIs.

A significant finding of the study was the identification of tinnitus as a notable AEFIs following COVID-19 vaccination. This aligns with the findings of other studies, such as the research conducted by Ahsanuddin et al. Their investigation, based on a comprehensive analysis of the FDA’s VAERS database, also identified a significant occurrence of otolaryngologic symptoms post COVID-19 vaccination, with tinnitus being notably prevalent (13). Specifically, they highlighted the significant reporting rates of tinnitus (PRR: 3.97, ROR: 3.98) following the COVID-19 vaccination, emphasizing them as higher than the background reporting rates in the database. In this study, as a result of analyzing PRR in the same way as in previous studies, tinnitus was found to be statistically significantly higher. Looking deeper into the potential mechanisms behind these symptoms, Ahsanuddin et al. suggested that the effects of the virus on the vestibulocochlear nerve could be a plausible cause for symptoms like tinnitus, deafness, and vertigo (13). Another hypothesis postulated the involvement of the middle ear’s epithelium, which, having a high expression of ACE2 receptors needed for the virus’s entry, might undergo inflammation or direct damage (23, 24). As such, it is speculated that the immunological response against spike proteins in COVID-19 vaccines might interact with cranial nerves and the middle ear, producing symptoms reminiscent of a viral infection. Drawing parallels with this study’s observations, the prominence of tinnitus as an AEFIs post COVID-19 vaccination cannot be understated. The findings resonate with previous research, such as the study by Dorney I et al., further emphasizing the importance of this particular AEFIs (25). While the precise mechanisms underpinning the development of tinnitus post-vaccination remain elusive, the accumulating evidence denotes a potential correlation between COVID-19 vaccines and the onset of tinnitus, necessitating more comprehensive clinical and mechanistic investigations.

The analysis of this study reveals a notable gender disparity in the frequency of otolaryngological AEFIs following COVID-19 vaccination, with a higher prevalence observed in females. This observation aligns with a cohort analysis conducted in Denmark and Iraq (26, 27), which also reported a higher frequency of AEFIs among females. This gender-based variation in response to vaccination, while not entirely understood, is becoming a salient feature in the growing body of research surrounding COVID-19 vaccines.

Systemic reactions, such as fever, have been more commonly reported among younger individuals following vaccination (28). However, contrasting findings from a study by Xiong et al. indicate that more severe outcomes, including serious AEFIs, permanent disabilities, hospitalizations, and death, were more frequently observed in older adults compared to younger adults aged between 18 and 64 years (29). Corroborating these findings, the analysis focusing on otolaryngological AEFIs similarly found a higher prevalence in older age groups. Specifically, within the cohort that received the completed primary series, there was a significant spike in AEFIs in the 50–64 age range. Additionally, data concerning the updated bivalent booster shot illustrated a more pronounced prevalence of AEFIs in individuals aged 65 and above. This accumulation of evidence suggests a distinct age-dependent variation in response to vaccination. This is further emphasized by studies showing that, compared to their younger counterparts, the older adult population seems to exhibit a diminished capacity to mount an effective immune response post-vaccination (30). For instance, Müller et al. demonstrated that older individuals had a reduced frequency of neutralizing antibodies following BNT162b2 vaccination relative to the younger demographic (31). Delving deeper into the causal factors underlying these age-related discrepancies necessitates further dedicated research.

The findings also shed light on differences across various vaccine manufacturers. Specifically, the rate of AEFIs following at least one dose of the Janssen vaccine was roughly twice as high as that observed with Pfizer and Moderna vaccines. Moderna, in turn, showed a slightly higher rate compared to Pfizer. This is consistent with previous reports suggesting that while local reactions may be more prevalent following mRNA vaccines (Pfizer and Moderna), systemic AEFIs, such as headache and fatigue, appear to be more prevalent following viral vector-based vaccines (e.g., Janssen) (32). These differences in AEFIs profiles among the vaccines are particularly noteworthy. They not only add depth to the understanding of the immune response triggered by different vaccine platforms but also highlight the need for personalized approaches to vaccination, taking into account factors such as age, gender, and individual health status.

In 2022, a study by Nguyen Dc et al. (33), involving 1,323 participants, demonstrated that the incidence of AEFIs following a booster vaccination was consistent with that of the first or second vaccination. However, this study has investigated that adverse reactions were more frequent after receiving the completed primary series (CPS) compared to the updated bivalent booster (UBB). Several factors might contribute to this observation. As individuals progress through the vaccination series, their adaptive immune response could become more refined and primed, potentially leading to fewer AEFIs after receiving the UBB compared to the CPS. Concurrently, there is the possibility of a reporting bias: individuals might initially be more vigilant in reporting AEFIs, viewing them as novel and anxiety-inducing. By the time they receive the booster shot, they might have grown accustomed to the vaccine and its potential side effects, resulting in decreased reporting. Despite these considerations, it remains crucial to acknowledge the limitations of the Vietnamese study due to its smaller sample size and a predominantly Asian participant demographic, which could introduce potential biases. Regardless, our findings hint at a degree of adaptability and tolerance developing in individuals as they progress through the vaccination series, serving as a reassuring indicator for public health campaigns aiming to boost vaccine uptake. Furthermore, it highlights the effectiveness of COVID-19 vaccination in containing SARS-CoV-2 spread and reducing the severity of COVID-19 disease, as well as the risk of developing long COVID (34).

This study, while extensive, has several inherent limitations that need to be acknowledged. The use of VAERS data, a passive and voluntary reporting system (35), likely leads to underreporting of AEFIs and may introduce reporting bias (36). Given the nature of this system, the quality and accuracy of the reported data may differ because one person can report multiple AEFIs. In addition, the study lacked a consistent denominator of administered doses, which restricted the capacity to accurately calculate incidence rates of AEFIs. Furthermore, this analysis predominantly focused on short-term post-vaccination effects, underscoring the need for longitudinal studies to assess potential long-term AEFIs among a more diverse and larger population. Finally, because the CDC only provides disaggregated information on gender, age, and manufacturer, only a univariate analysis could be conducted. Despite these limitations, these real-world, long-term descriptive studies are essential to further refine our understanding of the safety profile of COVID-19 vaccines. It is also imperative that future investigations corroborate reported AEFIs with additional clinical data and diagnostic tests to robustly establish causality.

## 5 Conclusion

The analysis contributes valuable insights into the landscape of otolaryngological AEFIs following COVID-19 vaccination, a relatively underexplored area in the current literature. It underscores the importance of vigilant post-vaccination surveillance and provides a foundation for further research aimed at elucidating the mechanisms behind these observations and informing safer and more effective vaccination strategies.

## Data availability statement

The original contributions presented in the study are included in the article/Supplementary material, further inquiries can be directed to the corresponding author.

## Ethic statement

The studies involving humans were approved by the institutional review board of K University (KYU-2023-09-002). The studies were conducted in accordance with the local legislation and institutional requirements. Written informed consent for participation was not required from the participants or the participants’ legal guardians/next of kin in accordance with the national legislation and institutional requirements.

## Author contributions

JS: Conceptualization, Data curation, Methodology, Resources, Software, Validation, Writing – review & editing, Writing – original draft. SS: Conceptualization, Data curation, Formal analysis, Methodology, Resources, Validation, Visualization, Writing – review & editing, Writing – original draft. JL: Methodology, Writing – original draft. HR: Methodology, Writing – original draft. J-YK: Conceptualization, Funding acquisition, Supervision, Validation, Writing – original draft, Writing – review & editing.

## Footnotes

^1^ https://vaers.hhs.gov/data.html

^2^ https://wonder.cdc.gov/vaers.html

^3^ https://covid.cdc.gov/covid-data-tracker/#datatracker-home

^4^ https://www.census.gov/newsroom/press-kits/2020/population-estimates-detailed.html
