# Supplementary material for: Otorhinolaryngologic complications after COVID-19 vaccination, vaccine adverse event reporting system (VAERS)
Source: Front Public Health. 2024 Jan 10;11:1338862. doi: 10.3389/fpubh.2023.1338862 (PMC10807421; doi:10.3389/fpubh.2023.1338862)
Supplement: Supplementary file 1 [file Data_Sheet_1.docx]

Supplementary Material

Otorhinolaryngologic Complications after COVID-19 Vaccination, Vaccine Adverse Event Reporting System(VAERS)

Jieun Shin^1,2^, Sung Ryul Shim^1,2^, Jaekwang Lee^3^ , Hyon Shik Ryu^3^ and Jong-Yeup Kim^1,2, 4,^*

^1^Department of Biomedical Informatics, College of Medicine, Konyang University, Daejeon 35365, Republic of Korea

^2^Konyang Medical data Research group-KYMERA, Konyang University Hospital, Daejeon 35365, Republic of Korea

^3^Department of Emergency Medicine, College of Medicine, Konyang University Hospital, Daejeon 35365, Republic of Korea

^4^Department of Otorhinolaryngology-Head and Neck Surgery, College of Medicine, Konyang University Hospital, Daejeon 35365, Republic of Korea

† Jieun Shin & Sung Ryul Shim contributed equally to this work as co-first author.

*** Correspondence:** Jong-Yeup Kim [jykim@kyuh.ac.kr](mailto:jykim@kyuh.ac.kr)

**Supplementary Table 1.** STROBE Statement—checklist of items that should be included in reports of observational studies.

|  | | Item No | Recommendation | Page  No |
| --- | --- | --- | --- | --- |
| **Title and abstract** | | 1 | (*a*) Indicate the study’s design with a commonly used term in the title or the abstract | 1 |
|  |  |  | (*b*) Provide in the abstract an informative and balanced summary of what was done and what was found | 1 |
| Introduction | | | | |
| Background/rationale | | 2 | Explain the scientific background and rationale for the investigation being reported | 2 |
| Objectives | | 3 | State specific objectives, including any prespecified hypotheses | 2 |
| Methods | | | | |
| Study design | | 4 | Present key elements of study design early in the paper | 3 |
| Setting | | 5 | Describe the setting, locations, and relevant dates, including periods of recruitment, exposure, follow-up, and data collection | 3-4 |
| Participants | | 6 | (*a*) *Cohort study*—Give the eligibility criteria, and the sources and methods of selection of participants. Describe methods of follow-up  *Case-control study*—Give the eligibility criteria, and the sources and methods of case ascertainment and control selection. Give the rationale for the choice of cases and controls  *Cross-sectional study*—Give the eligibility criteria, and the sources and methods of selection of participants | 3-4 |
|  |  |  | (*b*) *Cohort study*—For matched studies, give matching criteria and number of exposed and unexposed  *Case-control study*—For matched studies, give matching criteria and the number of controls per case |  |
| Variables | | 7 | Clearly define all outcomes, exposures, predictors, potential confounders, and effect modifiers. Give diagnostic criteria, if applicable | 4 |
| Data sources/ measurement | | 8* | For each variable of interest, give sources of data and details of methods of assessment (measurement). Describe comparability of assessment methods if there is more than one group | *3* |
| Bias | | 9 | Describe any efforts to address potential sources of bias | 4 |
| Study size | | 10 | Explain how the study size was arrived at | 5 |
| Quantitative variables | | 11 | Explain how quantitative variables were handled in the analyses. If applicable, describe which groupings were chosen and why | 4 |
| Statistical methods | | 12 | (*a*) Describe all statistical methods, including those used to control for confounding | 4 |
|  |  |  | (*b*) Describe any methods used to examine subgroups and interactions | 4 |
|  |  |  | (*c*) Explain how missing data were addressed |  |
|  |  |  | (*d*) *Cohort study*—If applicable, explain how loss to follow-up was addressed  *Case-control study*—If applicable, explain how matching of cases and controls was addressed  *Cross-sectional study*—If applicable, describe analytical methods taking account of sampling strategy |  |
|  |  |  | (*e*) Describe any sensitivity analyses |  |
| Results | | | | |
| Participants | 13* | (a) Report numbers of individuals at each stage of study—eg numbers potentially eligible, examined for eligibility, confirmed eligible, included in the study, completing follow-up, and analysed | | 5 |
|  |  | (b) Give reasons for non-participation at each stage | |  |
|  |  | (c) Consider use of a flow diagram | | 5 |
| Descriptive data | 14* | (a) Give characteristics of study participants (eg demographic, clinical, social) and information on exposures and potential confounders | | 5-6 |
|  |  | (b) Indicate number of participants with missing data for each variable of interest | |  |
|  |  | (c) *Cohort study*—Summarise follow-up time (eg, average and total amount) | | 5-6 |
| Outcome data | 15* | *Cohort study*—Report numbers of outcome events or summary measures over time | | *5-6* |
|  |  | *Case-control study—*Report numbers in each exposure category, or summary measures of exposure | |  |
|  |  | *Cross-sectional study—*Report numbers of outcome events or summary measures | |  |
| Main results | 16 | (*a*) Give unadjusted estimates and, if applicable, confounder-adjusted estimates and their precision (eg, 95% confidence interval). Make clear which confounders were adjusted for and why they were included | | 5-6 |
|  |  | (*b*) Report category boundaries when continuous variables were categorized | |  |
|  |  | (*c*) If relevant, consider translating estimates of relative risk into absolute risk for a meaningful time period | |  |
| Other analyses | 17 | Report other analyses done—eg analyses of subgroups and interactions, and sensitivity analyses | |  |
| Discussion | | | | |
| Key results | 18 | Summarise key results with reference to study objectives | | 6 |
| Limitations | 19 | Discuss limitations of the study, taking into account sources of potential bias or imprecision. Discuss both direction and magnitude of any potential bias | | 8 |
| Interpretation | 20 | Give a cautious overall interpretation of results considering objectives, limitations, multiplicity of analyses, results from similar studies, and other relevant evidence | | 8 |
| Generalisability | 21 | Discuss the generalisability (external validity) of the study results | | 6-7 |
| Other information | | | | |
| Funding | 22 | Give the source of funding and the role of the funders for the present study and, if applicable, for the original study on which the present article is based | | 9 |

*Give information separately for cases and controls in case-control studies and, if applicable, for exposed and unexposed groups in cohort and cross-sectional studies.

**Supplementary Table 2.** Medical Dictionary for Regulatory Activities concepts for 153 preferred terms as related to otorhinolaryngologic adverse events.

| Ears |  |
| --- | --- |
| ('CONDUCTIVE DEAFNESS [10010280]', 'DEAFNESS [10011878]', 'DEAFNESS BILATERAL [10052556]', 'DEAFNESS NEUROSENSORY [10011891]', 'DEAFNESS UNILATERAL [10048812]', 'HYPOACUSIS [10048865]', 'MIXED DEAFNESS [10027757]', 'SUDDEN HEARING LOSS [10061373]', 'TINNITUS [10043882]', 'ALLERGIC OTITIS MEDIA [10061557]', 'EAR INFECTION [10014011]', 'OTITIS EXTERNA [10033072]', 'OTITIS EXTERNA BACTERIAL [10065179]', 'OTITIS EXTERNA FUNGAL [10052557]', 'OTITIS MEDIA [10033078]', 'OTITIS MEDIA ACUTE [10033079]', 'OTITIS MEDIA BACTERIAL [10065176]', 'OTITIS MEDIA CHRONIC [10033081]', 'OTITIS MEDIA VIRAL [10065177]', 'MENIERE'S DISEASE [10027183]', 'VESTIBULAR NEURONITIS [10047393]', 'CERVICOGENIC VERTIGO [10075760]', 'DIZZINESS [10013573]', 'DIZZINESS EXERTIONAL [10013576]', 'DIZZINESS POSTURAL [10013578]', 'PERSISTENT POSTURAL-PERCEPTUAL DIZZINESS [10079170]', 'PROCEDURAL DIZZINESS [10066964]', 'VERTIGO [10047340]', 'VERTIGO CNS ORIGIN [10047343]', 'VERTIGO LABYRINTHINE [10047344]', 'VERTIGO POSITIONAL [10047348]') | |
| Nose |  |
| ('ACUTE SINUSITIS [10001076]', 'ALLERGIC SINUSITIS [10049153]', 'CHRONIC HYPERPLASTIC EOSINOPHILIC SINUSITIS [10071380]', 'CHRONIC SINUSITIS [10009137]', 'PARANASAL SINUS INFLAMMATION [10083836]', 'SINUSITIS [10040753]', 'SINUSITIS ASPERGILLUS [10051016]', 'SINUSITIS BACTERIAL [10060841]', 'SINUSITIS FUNGAL [10058678]', 'VIRAL SINUSITIS [10051513]', 'BACTERIAL RHINITIS [10065181]', 'FUNGAL RHINITIS [10065182]', 'RHINITIS [10039083]', 'RHINITIS ALLERGIC [10039085]', 'RHINITIS PERENNIAL [10039094]', 'RHINITIS SEASONAL [10039095]', 'VASOMOTOR RHINITIS [10047145]', 'VIRAL RHINITIS [10064948]', 'EPISTAXIS [10015090]', 'ANOSMIA [10002653]', 'NASAL POLYPS [10028756]', 'SNORING [10041235]', 'ADMINISTRATION SITE HYPERSENSITIVITY [10075102]', 'ALLERGY ALERT TEST POSITIVE [10075479]', 'ALLERGY PROPHYLAXIS [10066536]', 'ALLERGY TEST POSITIVE [10056352]', 'ALLERGY TO ANIMAL [10001742]', 'ALLERGY TO ARTHROPOD BITE [10058285]', 'ALLERGY TO ARTHROPOD STING [10058284]', 'ALLERGY TO CHEMICALS [10061626]', , 'ALLERGY TO METALS [10066414]', 'ALLERGY TO PLANTS [10054928]', 'ALLERGY TO SURGICAL SUTURES [10077279]', 'ALLERGY TO SYNTHETIC FABRIC [10076764]', 'ALLERGY TO VACCINE [10055048]', 'ALLERGY TO VENOM [10001751]', 'ALVEOLITIS ALLERGIC [10001890]', 'APPLICATION SITE HYPERSENSITIVITY [10063683]', 'ATOPY [10003645]', 'BRONCHIAL HYPERREACTIVITY [10066091]', 'CAFFEINE ALLERGY [10074895]', 'COCKROACH ALLERGY [10057643]', 'COLD URTICARIA [10009869]', 'CONTRAST MEDIA ALLERGY [10066973]', 'CROSS SENSITIVITY REACTION [10011411]', 'DERMATITIS ALLERGIC [10012434]', 'DERMATITIS CONTACT [10012442]', 'DOCUMENTED HYPERSENSITIVITY TO ADMINISTERED DRUG [10064372]', 'DOCUMENTED HYPERSENSITIVITY TO ADMINISTERED PRODUCT [10076470]', 'DRUG HYPERSENSITIVITY [10013700]', 'DRUG REACTION WITH EOSINOPHILIA AND SYSTEMIC SYMPTOMS [10073508]', 'DUST ALLERGY [10077439]', 'EYE ALLERGY [10015907]', 'FOOD ALLERGY [10016946]', 'HOUSE DUST ALLERGY [10057631]', 'HYPERSENSITIVITY [10020751]', 'HYPERSENSITIVITY MYOCARDITIS [10081004]', 'HYPERSENSITIVITY PNEUMONITIS [10081988]', 'HYPERSENSITIVITY VASCULITIS [10020764]', 'INFUSION RELATED HYPERSENSITIVITY REACTION [10082742]', 'INJECTION SITE HYPERSENSITIVITY [10022071]', 'IODINE ALLERGY [10052098]', 'LATEX ALLERGY [10056435]', 'MILK ALLERGY [10027633]', 'MITE ALLERGY [10077290]',, 'MULTIPLE ALLERGIES [10028164]', 'MYCOTIC ALLERGY [10052758]', 'NUTRITIONAL SUPPLEMENT ALLERGY [10084049]', 'ORAL ALLERGY SYNDROME [10068355]', 'POLYMERS ALLERGY [10086347]', 'RHINITIS ALLERGIC [10039085]', 'RUBBER SENSITIVITY [10039251]', 'SEASONAL ALLERGY [10048908]', 'TYPE I HYPERSENSITIVITY [10045240]', 'TYPE II HYPERSENSITIVITY [10054000]', 'TYPE III IMMUNE COMPLEX MEDIATED REACTION [10053614]', 'TYPE IV HYPERSENSITIVITY REACTION [10053613]', 'URTICARIA AQUAGENIC [10046739]', 'VACCINATION SITE HYPERSENSITIVITY [10068880]', 'APNOEA [10002974]', 'APNOEA NEONATAL [10002976]', 'APNOEA TEST [10053464]', 'APNOEA TEST ABNORMAL [10074913]', 'APNOEIC ATTACK [10002977]', 'CENTRAL SLEEP APNOEA SYNDROME [10007975]', 'INFANTILE APNOEA [10077321]', 'INFANTILE APNOEIC ATTACK [10065422]', 'NEONATAL APNOEIC ATTACK [10028922]', 'OBSTRUCTIVE SLEEP APNOEA SYNDROME [10029983]', 'SLEEP APNOEA SYNDROME [10040979]') | |
| Throat |  |
| ('ACUTE TONSILLITIS [10001093]', 'CHRONIC TONSILLITIS [10009152]', 'PERITONSILLITIS [10049592]', 'PHARYNGOTONSILLITIS [10049140]', 'TONSILLITIS [10044008]', 'TONSILLITIS BACTERIAL [10065235]', 'TONSILLITIS STREPTOCOCCAL [10044013]', 'VIRAL TONSILLITIS [10047480]', 'LARYNGITIS [10023874]', 'LARYNGITIS ALLERGIC [10064866]', 'LARYNGITIS BACTERIAL [10063070]', 'LARYNGITIS VIRAL [10023880]', 'RHINOLARYNGITIS [10057442]', 'SUBGLOTTIC LARYNGITIS [10076988]', 'VOCAL CORD DISORDER [10061581]', 'REFLUX LARYNGITIS [10067869]', 'CHRONIC THROAT CLEARING [10075070]', 'THROAT CLEARING [10080125]', 'EPIGLOTTITIS [10015030]', 'EPIGLOTTITIS HAEMOPHILUS [10015031]', 'EPIGLOTTITIS OBSTRUCTIVE [10015033]', 'ALLERGIC PHARYNGITIS [10050639]', 'FUNGAL PHARYNGITIS [10076516]', 'LARYNGOPHARYNGITIS [10061264]', 'NASOPHARYNGITIS [10028810]', 'OROPHARYNGITIS FUNGAL [10061891]', 'PHARYNGITIS [10034835]', 'PHARYNGITIS BACTERIAL [10057869]', 'PHARYNGITIS STREPTOCOCCAL [10034839]', 'VIRAL PHARYNGITIS [10047473]', 'HERPANGINA [10019936]', 'HERPES PHARYNGITIS [10066888]') | |

**Supplementary Table 3.** Major otorhinolaryngologic adverse events of COVID-19 Vaccination.

| **Adverse event** | **MedDRA term** |
| --- | --- |
| Hearing Loss |  |
|  | CONDUCTIVE DEAFNESS |
|  | DEAFNESS |
|  | DEAFNESS BILATERAL |
|  | DEAFNESS NEUROSENSORY |
|  | DEAFNESS UNILATERAL |
|  | HYPOACUSIS |
|  | MIXED DEAFNESS |
|  | SUDDEN HEARING LOSS |
| Tinnitus (Ringing in the ears) |  |
|  | TINNITUS |
| Ear Infections (Otitis Media) |  |
|  | ALLERGIC OTITIS MEDIA |
|  | EAR INFECTION |
|  | OTITIS EXTERNA |
|  | OTITIS EXTERNA BACTERIAL |
|  | OTITIS EXTERNA FUNGAL |
|  | OTITIS MEDIA |
|  | OTITIS MEDIA ACUTE |
|  | OTITIS MEDIA BACTERIAL |
|  | OTITIS MEDIA CHRONIC |
|  | OTITIS MEDIA VIRAL |
| Meniere's Disease |  |
|  | MENIERE'S DISEASE |
| Vestibular Neuronitis |  |
|  | VESTIBULAR NEURONITIS |
| Dizziness or Vertigo |  |
|  | CERVICOGENIC VERTIGO |
|  | DIZZINESS |
|  | DIZZINESS EXERTIONAL |
|  | DIZZINESS POSTURAL |
|  | PERSISTENT POSTURAL-PERCEPTUAL DIZZINESS |
|  | PROCEDURAL DIZZINESS |
|  | VERTIGO |
|  | VERTIGO CNS ORIGIN |
|  | VERTIGO LABYRINTHINE |
|  | VERTIGO POSITIONAL |
| Sinusitis |  |
|  | ACUTE SINUSITIS |
|  | ALLERGIC SINUSITIS |
|  | CHRONIC HYPERPLASTIC EOSINOPHILIC SINUSITIS |
|  | CHRONIC SINUSITIS |
|  | PARANASAL SINUS INFLAMMATION |
|  | SINUSITIS |
|  | SINUSITIS ASPERGILLUS |
|  | SINUSITIS BACTERIAL |
|  | SINUSITIS FUNGAL |
|  | VIRAL SINUSITIS |
| Rhinitis (Allergic and Non-allergic) |  |
|  | BACTERIAL RHINITIS |
|  | FUNGAL RHINITIS |
|  | RHINITIS |
|  | RHINITIS ALLERGIC |
|  | RHINITIS PERENNIAL |
|  | RHINITIS SEASONAL |
|  | VASOMOTOR RHINITIS |
|  | VIRAL RHINITIS |
| Epistaxis |  |
|  | EPISTAXIS |
| Anosmia |  |
|  | ANOSMIA |
| Nasal Polyps |  |
|  | NASAL POLYPS |
| Snoring or Difficulty Breathing through the Nose and Sleep Apnea |  |
|  | SNORING |
|  | APNOEA |
|  | CENTRAL SLEEP APNOEA SYNDROME |
|  | OBSTRUCTIVE SLEEP APNOEA SYNDROME |
|  | SLEEP APNOEA SYNDROME |
| Allergies |  |
|  | ADMINISTRATION SITE HYPERSENSITIVITY |
|  | ALLERGY ALERT TEST POSITIVE |
|  | ALLERGY PROPHYLAXIS |
|  | ALLERGY TEST POSITIVE |
|  | ALLERGY TO ANIMAL |
|  | ALLERGY TO ARTHROPOD BITE |
|  | ALLERGY TO ARTHROPOD STING |
|  | ALLERGY TO CHEMICALS |
|  | ALLERGY TO METALS |
|  | ALLERGY TO PLANTS |
|  | ALLERGY TO SURGICAL SUTURES |
|  | ALLERGY TO SYNTHETIC FABRIC |
|  | ALLERGY TO VACCINE |
|  | ALLERGY TO VENOM |
|  | ALVEOLITIS ALLERGIC |
|  | APPLICATION SITE HYPERSENSITIVITY |
|  | ATOPY |
|  | BRONCHIAL HYPERREACTIVITY |
|  | CAFFEINE ALLERGY |
|  | COCKROACH ALLERGY |
|  | COLD URTICARIA |
|  | CONTRAST MEDIA ALLERGY |
|  | CROSS SENSITIVITY REACTION |
|  | DERMATITIS ALLERGIC |
|  | DERMATITIS CONTACT |
|  | DOCUMENTED HYPERSENSITIVITY TO ADMINISTERED DRUG |
|  | DOCUMENTED HYPERSENSITIVITY TO ADMINISTERED PRODUCT |
|  | DRUG HYPERSENSITIVITY |
|  | DRUG REACTION WITH EOSINOPHILIA AND SYSTEMIC SYMPTOMS |
|  | DUST ALLERGY |
|  | EYE ALLERGY |
|  | FOOD ALLERGY |
|  | HOUSE DUST ALLERGY |
|  | HYPERSENSITIVITY |
|  | HYPERSENSITIVITY MYOCARDITIS |
|  | HYPERSENSITIVITY PNEUMONITIS |
|  | HYPERSENSITIVITY VASCULITIS |
|  | INFUSION RELATED HYPERSENSITIVITY REACTION |
|  | INJECTION SITE HYPERSENSITIVITY |
|  | IODINE ALLERGY |
|  | LATEX ALLERGY |
|  | MILK ALLERGY |
|  | MITE ALLERGY |
|  | MULTIPLE ALLERGIES |
|  | MYCOTIC ALLERGY |
|  | NUTRITIONAL SUPPLEMENT ALLERGY |
|  | ORAL ALLERGY SYNDROME |
|  | POLYMERS ALLERGY |
|  | RHINITIS ALLERGIC |
|  | RUBBER SENSITIVITY |
|  | SEASONAL ALLERGY |
|  | TYPE I HYPERSENSITIVITY |
|  | TYPE II HYPERSENSITIVITY |
|  | TYPE III IMMUNE COMPLEX MEDIATED REACTION |
|  | TYPE IV HYPERSENSITIVITY REACTION |
|  | URTICARIA AQUAGENIC |
|  | VACCINATION SITE HYPERSENSITIVITY |
| Tonsillitis |  |
|  | ACUTE TONSILLITIS |
|  | CHRONIC TONSILLITIS |
|  | PERITONSILLITIS |
|  | PHARYNGOTONSILLITIS |
|  | TONSILLITIS |
|  | TONSILLITIS BACTERIAL |
|  | TONSILLITIS STREPTOCOCCAL |
|  | VIRAL TONSILLITIS |
| Laryngitis |  |
|  | LARYNGITIS |
|  | LARYNGITIS ALLERGIC |
|  | LARYNGITIS BACTERIAL |
|  | LARYNGITIS VIRAL |
|  | RHINOLARYNGITIS |
|  | SUBGLOTTIC LARYNGITIS |
| Vocal Cord Polyps and Nodules |  |
|  | VOCAL CORD DISORDER |
| Laryngopharyngeal Reflux (Acid Reflux) |  |
|  | REFLUX LARYNGITIS |
|  | CHRONIC THROAT CLEARING |
|  | THROAT CLEARING |
| Epiglottitis |  |
|  | EPIGLOTTITIS |
|  | EPIGLOTTITIS HAEMOPHILUS |
|  | EPIGLOTTITIS OBSTRUCTIVE |
| Pharyngitis |  |
|  | ALLERGIC PHARYNGITIS |
|  | FUNGAL PHARYNGITIS |
|  | LARYNGOPHARYNGITIS |
|  | NASOPHARYNGITIS |
|  | OROPHARYNGITIS FUNGAL |
|  | PHARYNGITIS |
|  | PHARYNGITIS BACTERIAL |
|  | PHARYNGITIS STREPTOCOCCAL |
|  | VIRAL PHARYNGITIS |
|  | HERPANGINA |
|  | HERPES PHARYNGITIS |
